# Signal Transducers and Activators of Transcription 1 (STAT1), STAT2, and T Cells Mediate Interferon-Dependent Protection Against Neurobrucellosis

**DOI:** 10.1093/infdis/jiaf565

**Published:** 2025-11-12

**Authors:** Charles R Moley, Mostafa F N Abushahba, Bárbara Ponzilacqua-Silva, Irina Kochetkova, Christa D Jackson, Jerod A Skyberg

**Affiliations:** Department of Veterinary Pathobiology, College of Veterinary Medicine, University of Missouri, Columbia, Missouri, USA; Laboratory for Infectious Disease Research, University of Missouri, Columbia, Missouri, USA; Department of Veterinary Pathobiology, College of Veterinary Medicine, University of Missouri, Columbia, Missouri, USA; Laboratory for Infectious Disease Research, University of Missouri, Columbia, Missouri, USA; Department of Zoonoses, Faculty of Veterinary Medicine, Assiut University, Assiut, Egypt; Department of Veterinary Pathobiology, College of Veterinary Medicine, University of Missouri, Columbia, Missouri, USA; Laboratory for Infectious Disease Research, University of Missouri, Columbia, Missouri, USA; Department of Microbiology and Cell Biology, Montana State University, Bozeman, Montana, USA; Department of Microbiology and Cell Biology, Montana State University, Bozeman, Montana, USA; Department of Veterinary Pathobiology, College of Veterinary Medicine, University of Missouri, Columbia, Missouri, USA; Laboratory for Infectious Disease Research, University of Missouri, Columbia, Missouri, USA; Department of Microbiology and Cell Biology, Montana State University, Bozeman, Montana, USA

**Keywords:** *Brucella*, neurobrucellosis, T cell, interferon, STAT1, STAT2

## Abstract

**Background:**

Brucellosis is a significant zoonotic disease throughout the world. Human brucellosis patients develop flu-like symptoms and focal complications including arthritis and neurobrucellosis, which is the most morbid complication of *Brucella* infection.

**Methods:**

In this study, we employed murine models to uncover the role of T-cell–mediated immunity, interferons, and signal transducers and activators of transcription (STAT) signaling in the development of neurobrucellosis caused by *Brucella melitensis*.

**Results:**

Through adoptive transfer experiments, we discovered that T cells are recruited to the brains of *Brucella*-infected mice and are able to prevent central nervous system infection in an interferon-γ (IFN-γ)–dependent manner. Transferred T cells were also able to reduce established colonization of the brain by *Brucella*. In addition, we found that STAT1 plays a protective role against colonization of the brain by *Brucella* and the progression of neurobrucellosis, and that IFN-γ signaling is not entirely essential for these protective effects. While STAT2 deficiency alone did not affect *Brucella* burdens, a combined deficiency of STAT2 and the IFN-γ receptor led to elevated *Brucella* burdens in brains and blood, and a higher likelihood of developing neurologic symptoms relative to animals lacking the IFN-γ receptor alone.

**Conclusions:**

Our findings indicate that T cells and IFN signaling through both STAT1 and STAT2 play complex and important roles in protecting against bacterial colonization and development of neurologic symptoms following infection by *Brucella*.

## Teaser

Neurologic complications are the most morbid manifestation of human brucellosis. Here, we show T cells and interferons limit colonization of the brain by *Brucella* and the development of neurobrucellosis in a manner dependent on STAT1 and STAT2.

Brucellosis is a bacterial zoonosis caused by members of the *Brucella* genus that infects an estimated 2.1 million human patients per year [1]. *Brucella melitensis* is considered the primary human pathogen within this genus; however, human cases have been reported citing *Brucella abortus, Brucella suis, Brucella canis*, and *Brucella ceti* as causative agents [2, 3]. Human infection is primarily caused by direct contact with infected livestock or ingestion of unpasteurized dairy products, but can be transferred through aerosolization [2]. Although the bacterium poses a zoonotic risk worldwide, the disease is considered endemic throughout much of West Asia and North Africa, with the highest incidence in Syria, Kyrgyzstan, and Mongolia [4]. The most common symptoms of brucellosis include undulating fever, lethargy, chills, nausea, and headaches [4].

Neurobrucellosis is a potentially fatal complication that occurs when *Brucella* invades the central nervous system (CNS) and damages neural tissues, likely via induction of inflammatory responses [5, 6]. Mild sequelae include headaches, nausea, and dizziness; however, severe infections can lead to meningitis, lifelong neurologic disorders, and death [7]. The frequency of neurobrucellosis varies between studies [8, 9], but can be over 30% depending on the patient cohort [7].

Recently, we described the first murine model of neurobrucellosis in which *Brucella* crosses the blood-brain barrier (BBB), colonizes the brain, and impairs neurologic function, and demonstrated the protective effects of type I and II interferons (IFNs) against neurobrucellosis [10]. IFNs are a group of cytokines that signal through Janus kinase (JAK)/signal transducers and activators of transcription (STAT) pathways to communicate extracellular signals to the nucleus and initiate immune responses [11, 12]. IFNs can be broken down into 3 families: type I (IFN-α, IFN-β), type II (IFN-γ), and type III (IFN-λ). While all 3 families share some basic properties (induction by infection, immune modulation, transcriptional programming), there are a few key differences. Type I IFNs are secreted by many cell types (lymphocytes, fibroblasts, macrophages, etc.) and bind to a shared, heterodimeric surface receptor called the IFN-α/β receptor (IFNAR) that is ubiquitously expressed [11]. Type II IFN is secreted primarily by CD4^+^ T cells, natural killer cells, and CD8^+^ T cells, after which it signals through the IFN-γ receptor (IFNGR) [12]. The gene expression programs and signaling pathways of type III IFN closely resemble those of type I IFN; however, this IFN family is secreted primarily by epithelial cells and signals through the pairing of the IFN-λ receptor 1 (IFNLR1) subunit and the interleukin 10 receptor 2 (IL-10R2) subunit [11]. STAT1 (activated by all 3 IFN families) and STAT2 (activated only by type I and type III IFN) are the primary proteins responsible for IFN signaling [11, 12]. Here, we investigated the role of STAT signaling and T-cell–mediated immunity in IFN-mediated protection against neurobrucellosis.

## METHODS

### Bacterial Strains and Growth Conditions

All experiments with live *B. melitensis* were performed in biosafety level 3 facilities. *B. melitensis* 16M, obtained from Montana State University (Bozeman, Montana), was grown on *Brucella* agar (Ba) at 37°C (Becton Dickinson). Colonies were picked from Ba plates, and cultured in *Brucella* broth (Bb; Becton Dickinson) overnight at 37°C with shaking. *Brucella* concentration was estimated by measuring the optical density at 600 nm, and the inoculum was diluted to the appropriate concentration in sterile phosphate-buffered saline (sPBS) [13]. Actual viable titer was confirmed by serial dilution of inoculum onto Ba plates.

### Mice

Experiments were conducted using 6- to 12-week-old age- and sex-matched mice on a C57BL/6 background. C57BL/6J (WT), *Ifng^−/−^*, *Ifngr1^−/−^*, *Stat1^−/−^*, *Stat2^−/−^*, Ifnar1 floxed (*Ifnar1^fl/fl^*), and Lyz2Cre-expressing mice (Lyz2^Cre^) were obtained from the Jackson Laboratory (Bar Harbor, ME). *Ifngr1^−/−^* mice were intercrossed with *Stat1^−/−^* and *Stat2^−/−^* mice to generate *Ifngr1^−/−^*/*Stat1^−/−^* and *Ifngr1^−/−^*/*Stat2^−/−^* mice. Lyz2^Cre^ mice were intercrossed with *Ifnar1^fl/fl^* mice to generate Lyz2^Cre^*Ifnar1^fl/fl^* mice, and these animals were subsequently intercrossed with *Ifngr1^−/−^* animals to generate Lyz2^Cre^*Ifnar1^fl/fl^/Ifngr1^−/−^* mice. All mice were infected intranasally (IN) by first being anesthetized with 100 mg/kg ketamine and 10 mg/kg xylazine and then 20 µL of PBS containing 1 × 10^5^ colony-forming units (CFU) of *Brucella* was placed onto the anterior nares. All studies were conducted in accordance with University of Missouri Animal Care and Use Committee guidelines.

### Clinical Assessment of Neurobrucellosis

Mice were monitored for development of a head tilt, circling, or diminished proprioception. Animals displaying these signs were determined to have developed clinical neurobrucellosis. These recordings were made either at the conclusion of studies, or several times a week during a kinetic study.

### Adoptive Transfers

Spleens were harvested from naive mice and homogenized. Total T cells were purified magnetically using the EasySep Mouse T-cell isolation kit from StemCell Technologies. Following purification, cells were resuspended in sPBS and 3–5 × 10^6^ T cells were injected intraperitoneally into recipient animals [14].

### Measurement of Bacterial Burdens and Cytokines in Tissues

After infection, mice were euthanized, and blood, spleens, lungs, and/or brains were harvested. Tissues were homogenized mechanically in sPBS. A series of 10-fold dilutions were performed in triplicate in sPBS and plated onto Ba. Plates were incubated for 3–4 days at 37°C/5% CO_2_, colonies were enumerated, and the number of CFUs/tissue was calculated (brains and blood were incubated up to 7 days).

### Brain Histology

Prior to removal of brain, mice were exsanguinated and 2–3 mL of sPBS was perfused through the left ventricle to reduce blood contamination. Brains were cut sagittally with 1 hemisphere being homogenized for bacterial burden analysis, while the other was fixed in 10% buffered zinc formalin and embedded in paraffin. Brains were cut into sections (5 μm) and placed on glass slides. Sections were stained with hematoxylin and eosin (H&E) and covered with a coverslip. Immunohistochemistry was performed by IDEXX BioAnalytics (Columbia, MO). Brains were sectioned as described above. Enzyme immunohistochemical staining for CD4 and CD8 was performed on deparaffinized sections. Slides were lightly counterstained with hematoxylin, and coverslips added with permanent mounting medium.

### Brain Pathology Scoring

H&E slides were scored in a blinded manner. Meninges were assessed for hemorrhaging, cuffing, and subdural inflammation, and assigned a score of 0 (no lesions), 1 (single lesion), 2 (2–3 lesions), 3 (4–6 lesions), or 4 (>6 lesions or diffuse lesions). Brains were also assessed for hemorrhaging, gliosis, vacuolization, astrocyte accumulation, vascular damage, and necrosis, and assigned a score of 0 (no lesions), 1 (lesion in 1 part of brain), 2 (lesions in 2 parts of brain), 3 (lesions in 3–4 parts of brain), or 4 (inflammation/damage in all parts of brain).

### Statistical Analysis

Statistical analysis of the difference between 2 mean values was conducted using a 2-tailed Student *t* test with significance set at *P* ≤ .05, while comparisons of ≥ 3 mean values were done using ANOVA, followed by Tukey test with significance set at *P* ≤ .05. The development of neurologic signs over time was analyzed by log-rank analysis on incidence curves, while Fisher exact test was used to compare incidence of neurologic signs at the end of studies. The Mann-Whitney *U* test was used to compare pathology scores. All error bars display standard deviation, and the number of experimental repeats and n values are provided in figure legends.

## RESULTS

### Adoptive T-Cell Transfer Prevents *Brucella* From Further Disseminating and Reduces Previously Established CNS Infection in *Rag2^−/−^/Il2rg^−/−^* Mice Following Pulmonary Infection

**Figure 1.**
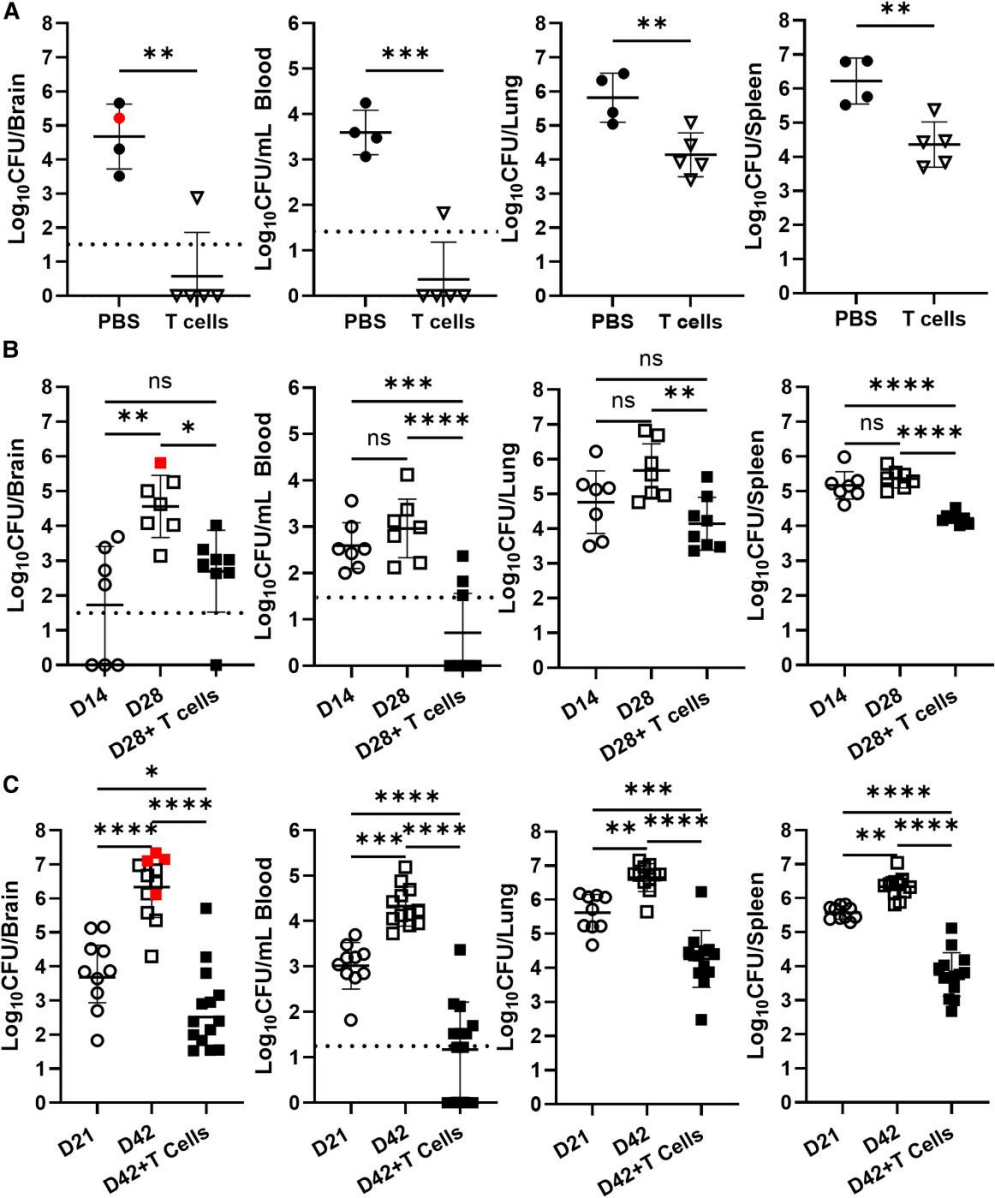
Adoptive T-cell transfer during *Brucella* infection protects against further bacterial dissemination and reduces previously established infection in *Rag2^−/−^/Il2rg^−/−^* mice. *A*, *Rag2^−/−^/Il2rg^−/−^* mice (n = 4–5) were administered either naive T cells or PBS intraperitoneally 1 day prior to IN infection with 10^5^ CFUs of *B. melitensis*; 28 days postinfection, *Brucella* burdens in the brain, blood, lung, and spleen were determined. *B*, *Rag2^−/−^/Il2rg^−/−^* mice (n = 7–8) were infected IN with 10^5^ CFUs of *B. melitensis*. Fourteen days postinfection, mice were either sacrificed for examination of bacterial burden (D14) or administered either PBS (D28) or naive T cells (D28+ T cells); 28 days postinfection, *Brucella* burdens in the brain, blood, lung, and spleen were determined. *C*, *Rag2^−/−^/Il2rg^−/−^* mice (n = 10–14) were infected IN with 10^5^ CFUs of *B. melitensis*. Twenty-one days postinfection, mice were either sacrificed for examination of bacterial burden (D21) or administered either PBS (D42) or naive T cells (D42+ T cells); 42 days postinfection, *Brucella* burdens in the brain, blood, lung, and spleen were determined (due to plate contamination, CFU data were unavailable from 1 lung sample in each group). *A*, Data are from 1 experiment. *B* and *C*, Data are combined from 2 experiments. *P < .05, **P < .01, ***P < .001, ****P < .0001. Red symbols in brain CFU graphs indicate mice with neurologic signs at the time of necropsy. Dashed lines indicate limit of detection. Abbreviations: CFU, colony-forming unit; IN, intranasal; ns, not significant; PBS, phosphate-buffered saline.

Previously, we found *B. melitensis* was able to colonize the brain and cause neurologic complications in *Rag2^−/−^/Il2rg^−/−^* mice, which lack B cells, T cells, and innate lymphoid cells [10]. To determine if T cells alone could prevent brain colonization in these animals, naive splenic T cells from WT mice or PBS were adoptively transferred into *Rag2^−/−^/Il2rg^−/−^* mice 1 day prior to infection with *B. melitensis*. At 28 days following challenge, we found that when compared to mice that received PBS, mice that received T cells displayed 100–1000-fold lower bacterial counts within the brain, blood, lung, and spleen (Figure 1*A*). To determine if transfer of T cells could halt an established infection, we infected *Rag2^−/−^/Il2rg^−/−^* mice with *B. melitensis* and on day 14 postinfection, transferred either PBS or naive WT splenic T cells. A cohort of mice was also sacrificed on day 14 postinfection and CFUs were measured to establish a baseline of infection prior to T-cell transfer. On day 28, all remaining mice were sacrificed and CFU levels were determined. T-cell–recipient mice not only had lower amounts of CFUs in all examined organs when compared to mice that received PBS (Figure 1*B*), but *Brucella* burdens were significantly reduced in blood and spleens of T-cell–recipient mice when compared to baseline levels 14 days postinfection (Figure 1*B*). These results demonstrate the ability of adoptively transferred T cells to prevent progressive infection by *Brucella*, as well as reduce established infection in blood and spleen.

To determine if transfer of T cells could reduce established infection of the brain, an experiment was designed in which *Rag2^−/−^/Il2rg^−/−^* mice were infected with *B. melitensis* and 21 days later animals received PBS or naive T cells. Six weeks postinfection mice were euthanized and organs harvested. Similar to our previous results, T-cell transfer 21 days after infection was sufficient in preventing progression of infection in all tissues examined (Figure 1*C*). Additionally, T-cell transfer into chronically infected mice significantly reduced previously established infection in brain, blood, lung, and spleen when compared to baseline levels present at the time of T-cell transfer (Figure 1*C*). These findings show that transfer of T cells can both prevent and reduce established colonization of systemic tissues and the brain during *Brucella* infection in *Rag2^−/−^/Il2rg^−/−^* mice.

### Protective Effects Conferred by Adoptively Transferred T Cells Are IFN-γ Dependent

**Figure 2.**
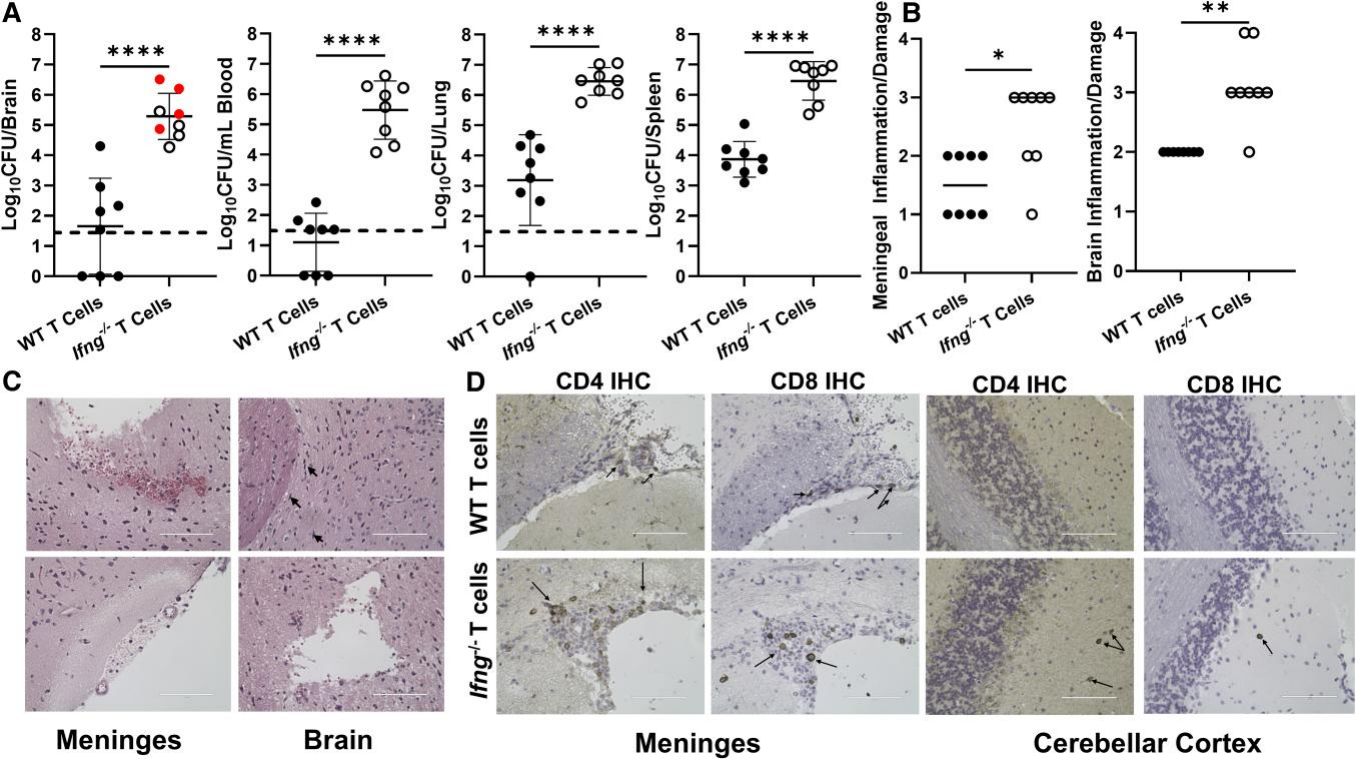
Protective effects conferred by adoptively transferred T cells are IFN-γ dependent. *A–D*, *Rag2^−/−^/Il2rg^−/−^* mice (n = 8) were administered T cells from either WT or *Ifng^−/−^* mice 1 day prior to intranasal infection with 10^5^ CFUs of *B. melitensis*; 28 days postinfection, *Brucella* burdens in the brain, blood, lung, and spleen were determined (*A*) and brains were sagittally sectioned for histology and scored (*B–D*). *B*, Inflammation, hemorrhaging, gliosis, vacuolization, astrocyte accumulation, vascular damage, and necrosis were scored within meninges and brains, as described in the “Methods” section. *C*, Left (top and bottom), representative hematoxylin and eosin stained images showing damaged meninges and hemorrhaging with surrounding vacuolization and necrosis. Top right, representative image showing rod cells indicating gliosis (marked with arrows) and subcortical vacuolization/necrosis. Bottom right, representative image of a subcortical lesion with surrounding vacuolization. *D*, Immunohistochemistry was performed to detect CD4^+^ and CD8^+^ T cells in the midbrain meninges (left) and parenchyma of the cerebellar cortex (right). Arrows indicate T-cell infiltration. *C* and *D*, White scale bars denote 100 µm. Data are from 1 experiment. *P < .05, **P < .01, ****P < .0001. Red symbols in brain CFU graph indicate mice with neurologic signs at the time of necropsy. Dashed lines indicate limit of detection. Abbreviations: CFU, colony-forming unit; IFN-γ, interferon-γ; IHC, immunohistochemistry; WT, wild type.

Previously we showed IFN-γ limits colonization of the brain by *Brucella* [10]. To determine the role of IFN-γ in the protective effects of T cells, *Rag2^−/−^/Il2rg^−/−^* mice received naive splenic T cells from either WT or *Ifng^−/−^* mice 1 day prior to infection with *B. melitensis*. On day 28 postinfection, 4 of 8 of mice that received T cells from *Ifng^−/−^* mice displayed overt neurologic signs compared to 0 of 8 mice that received WT T cells (Figure 2*A*). *Brucella* burdens within blood and brains of *Ifng^−/−^* T-cell recipients were approximately 10 000-fold higher when compared to those of WT T-cell recipients and burdens within spleen and lung were approximately 1000-fold higher in *Ifng^−/−^* T-cell recipients (Figure 2*A*). Mice that received *Ifng^−/−^* T cells also had higher levels of meningeal inflammation and other lesions within the brain such as hemorrhaging, gliosis, vacuolization, and necrosis than did WT T-cell recipients (Figure 2*B* and 2*C*). Immunohistochemistry revealed both CD4^+^ and CD8^+^ T cells from either WT or *Ifng^−/−^* animals were able to reach the meninges of *Rag2^−/−^/Il2rg^−/−^* mice after infection (Figure 2*D*). CD4^+^ and CD8^+^ T cells from *Ifng^−/−^* mice were also found in the parenchyma of the cerebellar cortex of infected recipients (Figure 2*D*).

**Figure 3.**
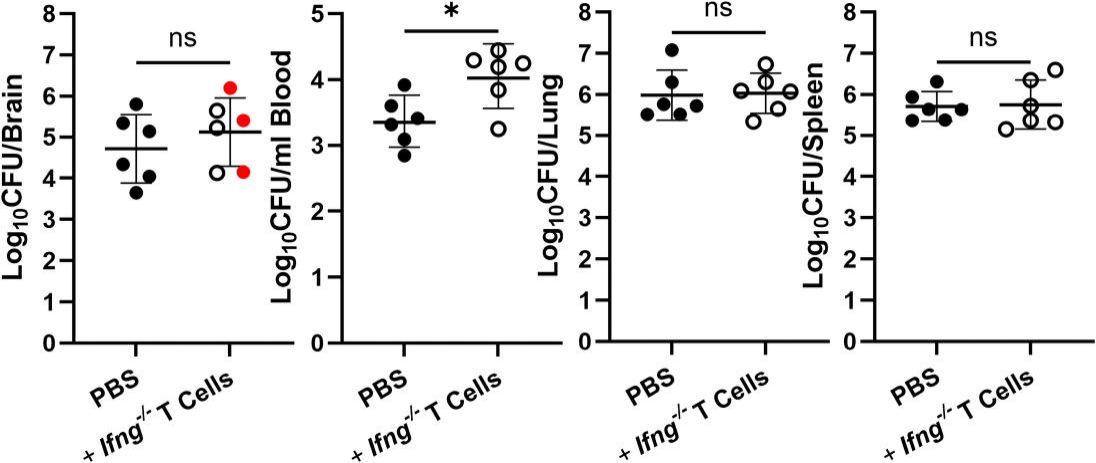
Transfer of T cells lacking the ability to produce IFN-γ enhances bacteremia. *Rag2^−/−^/Il2rg^−/−^* mice (n = 6) were administered either PBS or naive T cells from *Ifng^−/−^* mice 1 day prior to intranasal infection with 10^5^ CFUs of *B. melitensis*; 28 days postinfection, *Brucella* burdens in the brain, blood, lung, and spleen were determined. Data are from 1 experiment. *P < .05. Red symbols in brain CFU graph indicate mice with neurologic signs at the time of necropsy. Abbreviations: CFU, colony-forming unit; IFN-γ, interferon-γ; ns, not significant; PBS, phosphate-buffered saline.

In addition, in an experiment where we transferred PBS or naive splenic *Ifng^−/−^* T cells 1 day before infection, we found 3 of 6 *Rag2^−/−^/Il2rg^−/−^* mice that received *Ifng^−/−^* T cells developed neurologic complications by day 28 postinfection compared to 0 of 6 PBS-treated controls (Figure 3). *Rag2^−/−^/Il2rg^−/−^* mice that received *Ifng^−/−^* T cells also had higher levels of bacteremia than did PBS-treated controls (Figure 3). Collectively, these findings indicate protection conferred by transferred T cells is IFN-γ dependent, and that in the absence of IFN-γ, T cells promote bacteremia.

### STAT1 Signaling Protects Against Neurobrucellosis in a Manner not Entirely Dependent on IFN-γ Signaling

**Figure 4.**
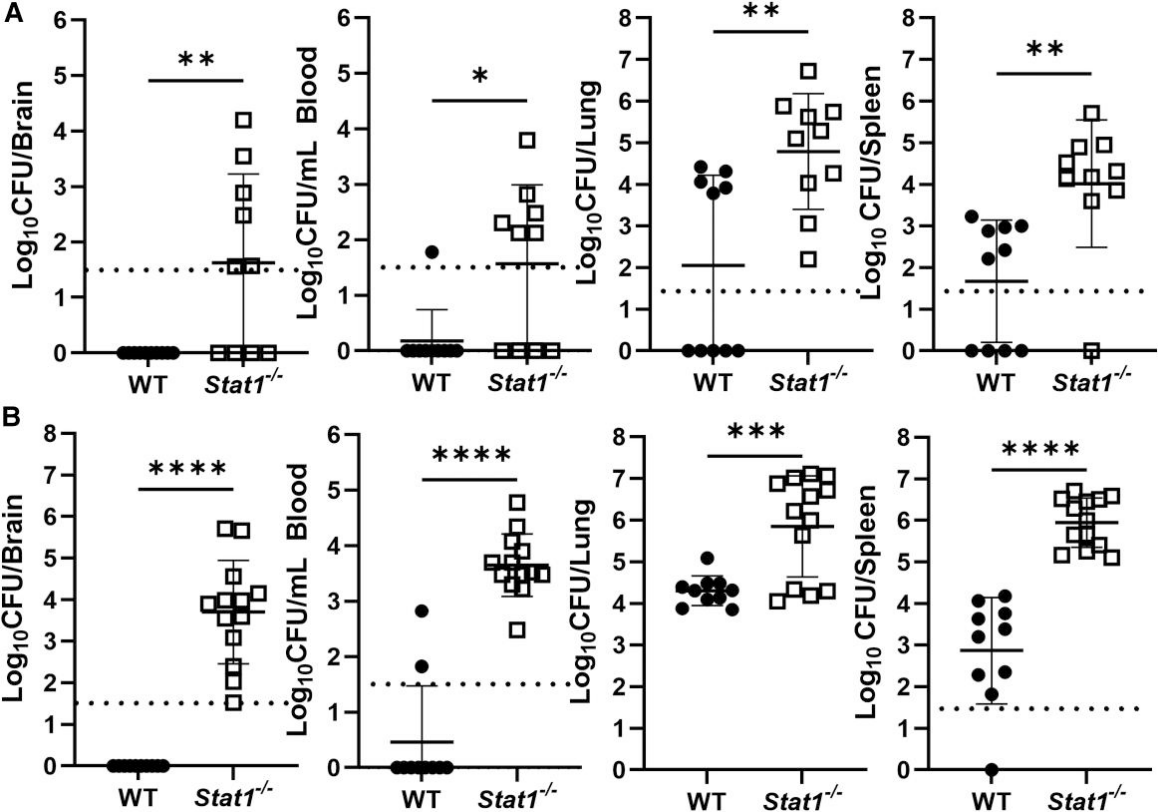
STAT1 signaling protects against colonization by *Brucella* following pulmonary infection. WT and *Stat1^−/−^* mice (n = 10–13) were infected intranasally with 10^5^ CFUs of *B. melitensis*; 14 (*A*) or 21 (*B*) days postinfection, *Brucella* burdens in the brain, blood, lung, and spleen were determined. Data are combined from 2 experiments. *P < .05, **P < .01, ***P < .001, ****P < .0001. Dashed lines indicate limit of detection. Abbreviations: CFU, colony-forming unit; STAT, signal transducers and activators of transcription; WT, wild type.

Type I, II, and III IFN signal through STAT1 [11, 12]; however, the role of STAT1 in *Brucella* infection is unknown. Therefore, we infected STAT1-deficient mice (*Stat1^−/−^*) along with WT animals, and examined CFUs in brain, blood, lungs, and spleen. *Stat1^−/−^* mice displayed significantly higher levels of *B. melitensis* within all organs examined when compared to WT mice at both 14 days (Figure 4*A*) and 21 days (Figure 4*B*) after infection, indicating STAT1 signaling protects against colonization by *Brucella*.

**Figure 5.**
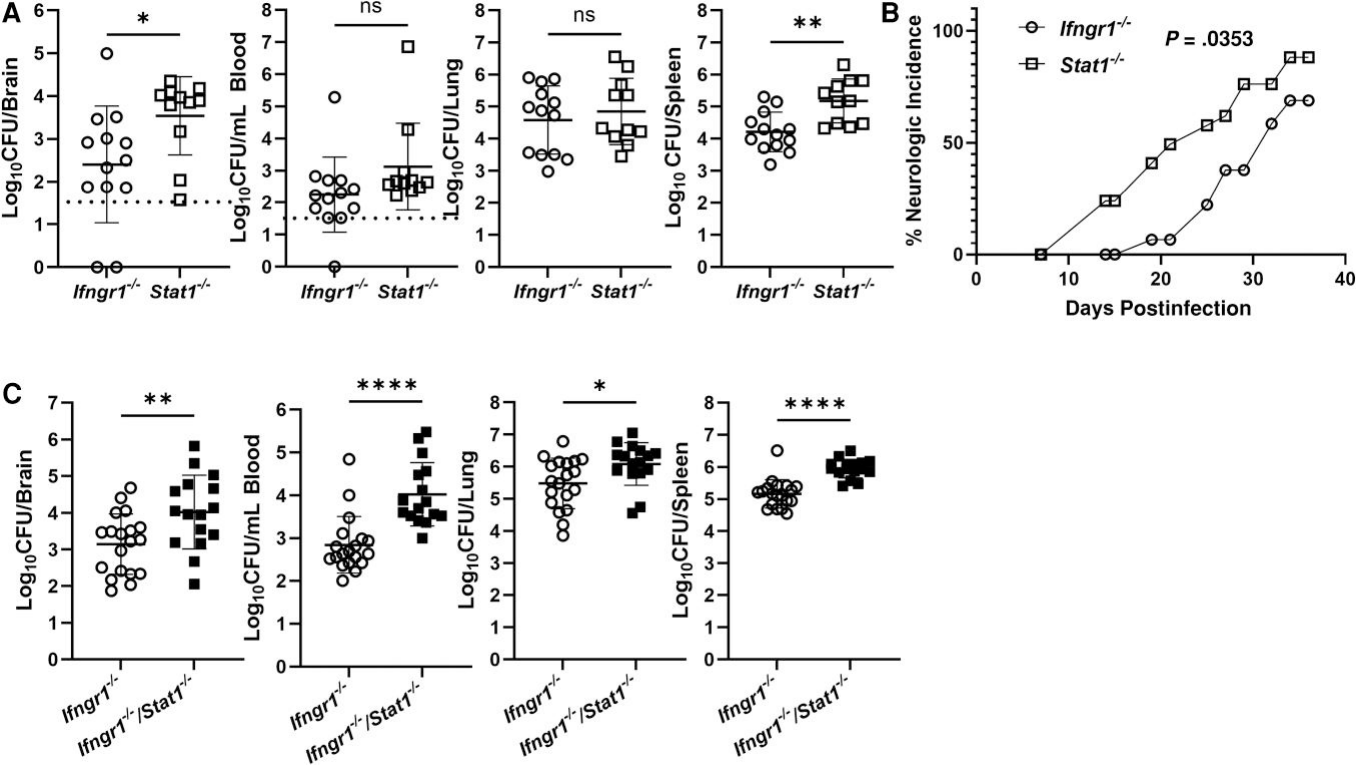
Protective effects conferred by STAT1 signaling against neurobrucellosis can be independent of type II Interferon. *A*, *Ifngr1^−/−^* and *Stat1^−/−^* mice (n = 11–13) were infected IN with 10^5^ CFUs of *B. melitensis*; 14 days postinfection, *Brucella* burdens in the brain, blood, lung, and spleen were determined. *B*, *Ifngr1^−/−^* and *Stat1^−/−^* mice (n = 18–26) were infected IN with 10^5^ CFUs of *B. melitensis* and signs of neurologic disease (head tilt, circling, diminished proprioception) were recorded over time. *C*, *Ifngr1^−/−^* and *Ifngr1^−/−^/Stat1^−/−^* mice (n = 16–19) were infected IN with 10^5^ CFUs of *B. melitensis*; 17 days postinfection, *Brucella* burdens in the brain, blood, lung, and spleen were determined. *A* and *C*, Data in each were combined from 2 experiments. *B*, Data are from 1 experiment. *P < .05, **P < .01, ****P < .0001. Dashed lines indicate limit of detection. Abbreviations: CFU, colony-forming unit; IN, intranasal; ns, not significant; STAT, signal transducers and activators of transcription.

To examine the requirement of IFN-γ signaling in STAT1-mediated protection against neurobrucellosis, we first compared the relative abilities of STAT1 and IFN-γ to protect against *Brucella*. *Stat1^−/−^* mice displayed significantly higher amounts of *Brucella* in brain and spleen when compared to *Ifngr1^−/−^* mice on day 14 postinfection (Figure 5*A*). To characterize the timeline of clinical disease, we performed a kinetic study by infecting and monitoring *Stat1^−/−^* and *Ifngr1^−/−^* mice for neurologic signs. We noted that *Stat1^−/−^* mice developed neurologic signs (head tilt, circling, incoordination, prolonged recumbency) at a greater rate than *Ifngr1^−/−^* mice over the 39-day infection (Figure 5*B*). This indicates the protective effects of STAT1 signaling are most likely not due to IFN-γ alone, but instead a combination of both IFN-γ and type I IFNs.

To confirm that STAT1 signaling can protect the host in an IFN-γ–independent manner, we generated *Ifngr1^−/−^/Stat1^−/−^* mice and infected them along with *Ifngr1^−/−^* animals. For this experiment, animals were euthanized 17 days postinfection as some *Ifngr1^−/−^/Stat1^−/−^* mice developed clinical signs that met qualifications for a humane end point by this time. When examining bacterial burdens in brain, blood, lung, and spleen, we found mice lacking both IFNGR1 and STAT1 had significantly increased amounts of bacteria in all organs when compared to mice lacking IFNGR1 alone (Figure 5*C*). These results confirm STAT1 signaling can play a protective role against the progression of *Brucella* infection in the brain and systemic tissues independently of IFN-γ.

### Type I IFN and STAT2 Signaling Alone Do Not Play a Significant Role in Protection Against *Brucella* Infection Following Pulmonary Infection

**Figure 6.**
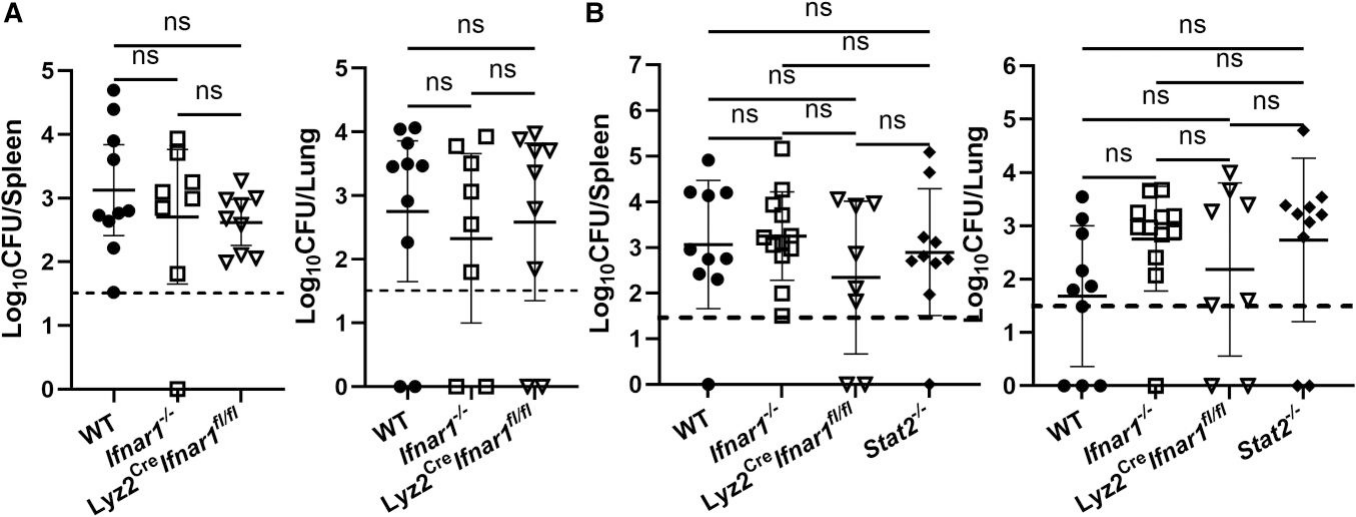
Type I IFN and STAT2 signaling alone do not alter tissue colonization following pulmonary *Brucella* infection. *A*, WT, *Ifnar1^−/−^*, and Lyz2^Cre^*Ifnar1^fl/fl^* mice (n = 8–10) were infected IN with 10^5^ CFUs of *B. melitensis* and 14 days postinfection *Brucella* burdens in lung and spleen were determined. *B*, WT, *Stat2^−/−^*, *Ifnar1^−/−^*, and Lyz2^Cre^*Ifnar1^fl/fl^* mice (n = 8–12) were infected IN with 10^5^ CFUs of *B. melitensis* and 28 days postinfection, *Brucella* burdens in lung and spleen were determined. Data are combined from 2 experiments. Dashed lines indicate limit of detection. Abbreviations: CFU, colony-forming unit; IFN, interferon; IN, intranasal; ns, not significant; STAT, signal transducers and activators of transcription; WT, wild type.

While IFN-γ is known to protect against both systemic and focal *Brucella* infection [15, 16], the role of type I IFNs in defense against *Brucella* is nebulous [10, 17, 18]. Type I IFNs can control *Brucella* infection in cultured macrophages by activating guanylate-binding proteins [17], while mice lacking receptors for type I IFNs (*Ifnar1^−/−^*) are resistant to splenic colonization by *Brucella* and display enhanced IFN-γ production following systemic infection [18]. We hypothesized that myeloid-specific IFNAR1 signaling could potentially protect against *Brucella* infection via guanylate-binding protein activation. To test this hypothesis, Lyz2^Cre^-expressing mice were crossed to mice with floxed *Ifnar1* alleles (*Ifnar1^fl/fl^*) to generate mice (Lyz2^Cre^*Ifnar1^fl/fl^*) lacking type I IFN signaling specifically in myeloid cells (macrophages, microglia, etc.). These mice, along with WT and *Ifnar1^−/−^* mice, were then infected with *B. melitensis*; however, no significant differences in CFU levels were found in either spleens or lungs at 14 days postinfection (Figure 6*A*). We also examined CFU levels 28 days postinfection and included *Stat2^−/−^* mice in these experiments but again found no significant differences between genotypes (Figure 6*B*). Combined, these results indicate STAT2 and myeloid-specific type I IFN signaling are not playing a significant role in protection against pulmonary *Brucella* infection at these time points.

### STAT2 Signaling in the Absence of Type II IFN Is Protective Against Neurobrucellosis

**Figure 7.**
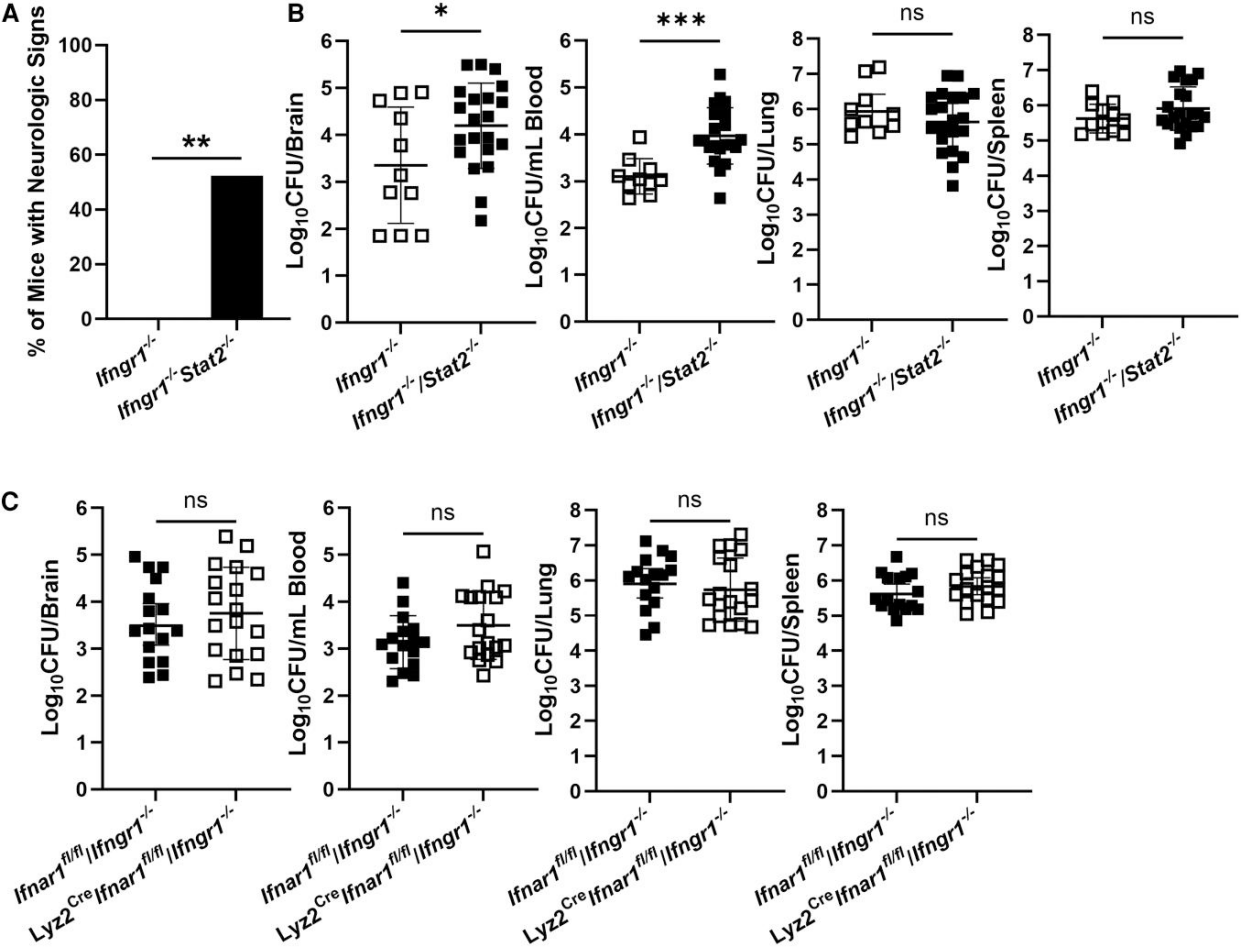
STAT2 signaling in the absence of type II interferon signaling is protective against neurobrucellosis *A* and *B*, *Ifngr1^−/−^* and *Ifngr1^−/−^/Stat2^−/−^* mice (n = 12–21) were infected IN with 10^5^ CFUs of *B. melitensis*; 17 days postinfection, the incidence of neurologic signs was recorded (*A*) and *Brucella* burdens in the brain, blood, lung, and spleen were measured (*B*) (due to plate contamination, CFU data was unavailable for 1 brain, 2 blood, and 2 lung samples in the *Ifngr1^−/−^* group). *C*, *Ifnar1^fl/fl^*/*Ifngr1^−/−^* (control) and Lyz2^Cre^*Ifnar1^fl/fl^*/*Ifngr1^−/−^* mice (n = 16–18) were infected IN with 10^5^ CFUs of *B. melitensis*; 17 days postinfection, *Brucella* burdens in the brain, blood, lung, and spleen were determined. *P < .05, **P < .01, ***P < .001. Data are combined from 2–3 experiments. Abbreviations: CFU, colony-forming unit; IN, intranasal; ns, not significant.

While both type I and type II IFN activate STAT1, STAT2 activation is limited to type I and type III IFN [11, 12]. As demonstrated in Figure 6, mice lacking STAT2 or type I IFN signaling do not display altered susceptibility to pulmonary infection by *Brucella*. However, we previously found mice lacking both type I and type II IFN signaling were more susceptible to *Brucella* than mice lacking type II IFN alone [10]. To determine if STAT2 could play a compensatory, protective role in the absence of IFN-γ, we infected *Ifngr1^−/−^* and *Ifngr1^−/−^/Stat2^−/−^* mice. Strikingly, 17 days after infection 11 of 21 *Ifngr1^−/−^/Stat2^−/−^* mice were exhibiting overt neurologic signs while 0 of 12 of the *Ifngr1^−/−^* mice showed such signs (Figure 7*A*). In addition, *Ifngr1^−/−^/Stat2^−/−^* mice had significantly higher bacterial burdens in brain and blood when compared to *Ifngr1^−/−^* mice; however, no significant differences in *Brucella* burden were noted in spleen or lung (Figure 7*B*). This indicates STAT2 may play a tissue-specific role in controlling *Brucella* infection in the absence of IFNGR signaling. Interestingly, deficiency of IFNAR1 signaling in myeloid cells did not further enhance the susceptibility of *Ifngr1^−/−^* mice to *Brucella* infection (Figure 7*C*), suggesting type I IFN/STAT2 signaling in nonmyeloid cells mediates a compensatory, protective role in mice lacking IFNGR1.

## DISCUSSION

CD4^+^ and CD8^+^ T cells have been noted in brains of human brucellosis patients and implicated in the pathogenesis of disease [19]; however, no studies have been published analyzing these cells in an animal model of neurobrucellosis. Here, by adoptive transfer studies we show naive WT T cells are able to prevent infection of the CNS by *Brucella* (Figure 1*A*), indicating T cells are sufficient in preventing colonization of the brain. In addition, WT T cells were able to reduce established infection of the CNS by *Brucella* (Figure 1*C*), indicating T cells can protect against infection even after *Brucella* has entered the brain. Transferred CD4^+^ and CD8^+^ T cells from *Ifng^−/−^* mice were also able to reach the brains of *Rag2^−/−^/Il2rg^−/−^* recipients but were unable to reduce infection of the brain (Figure 2 and Figure 3). In addition, *Rag2^−/−^/Il2rg^−/−^* mice that received *Ifng^−/−^* T cells had enhanced bacteremia and were more likely to develop neurologic signs than PBS-treated controls (Figure 3). Therefore, in the absence of IFN-γ signaling, T cells promote bacteremia and may contribute to development of neurologic complications, although additional studies would be required to confirm this.

Both type I and type II IFN signal through STAT1 [11, 12]. We found STAT1 confers protection against *Brucella* (Figure 4), especially in defense against bacteremia and CNS infection. Mice lacking both STAT1 and IFNGR1 also displayed significantly higher bacterial burdens in brains, blood, lungs, and spleen than mice lacking IFNGR1 alone (Figure 5*C*). These data suggest both IFN-γ and type I IFN contribute to STAT1-mediated control of infection. However, we found mice lacking only type I IFN signaling did not display an altered ability to control infection (Figure 7), indicating type I IFN may play a compensatory role in protecting against *Brucella* when type II IFN signaling is absent. Type I IFNs can signal through STAT1 homodimers or STAT1:STAT2 heterodimers [20]. We found *Ifngr1^−/−^/Stat2^−/−^* mice developed neurologic complications and had higher levels of *Brucella* in brain and blood when compared mice to lacking IFNGR alone (Figure 7*A* and 7*B*). This indicates signaling through STAT2 in the absence of IFN-γ plays a compensatory protective role against neurobrucellosis. These findings, coupled with our previous results [10], confirm that in the absence of IFN-γ, type I IFN plays a protective role against *Brucella* by signaling through both STAT1 and STAT2. As IFN-λ can also signal through STAT1 and STAT2, in the future the role of IFN-λ in development of neurobrucellosis should be investigated.

Myeloid cells are considered the main reservoir of *Brucella* in vivo [21], and *Ifnar1^−/−^* macrophages are more susceptible to *Brucella* in vitro [17]. However, we found similar *B. melitensis* burdens in brain, blood, lung, and spleens of *Ifngr1^−/−^* mice and in *Ifngr1^−/−^* mice that also lacked myeloid IFNAR1 signaling (Figure 7*C*), indicating IFNAR1 signaling in nonmyeloid cells contributes to protection conferred by type I IFN and STAT2 against neurobrucellosis. Type I IFNs can help maintain BBB integrity [22] and restrict lung permeability during bacterial infection to restrain bacteremia [23]. These findings are of note, as we found higher levels of *Brucella* in blood and brain, but not lungs, of *Ifngr1^−/−^*/*Stat2^−/−^* mice relative to *Ifngr1^−/−^* animals (Figure 7*B*). Similarly, in our previous study we found *Ifngr1^−/−^*/*Ifnar1^−/−^* mice had elevated levels of *Brucella* in blood, brain, and spleen, but not lungs, when compared to *Ifngr1^−/−^* mice [10]. Collectively, these data indicate type I IFN signaling through STAT2 in epithelial/endothelial cells of the lung and/or brain could protect against *Brucella* by limiting bacteremia and/or invasion of the brain; however, future studies would be necessary to confirm this.

A limitation of our study is that only immunodeficient mice develop neurologic complications following *Brucella* infection. Thus, in the future our work could be complemented by in vitro studies using wild-type cells. In vitro studies have indicated *Brucella*-infected monocytes are able to traverse human brain microvascular endothelial cells (HBMECs) and therefore potentially cross the BBB to access the CNS [24]. Therefore, the role of IFNs and STAT signaling in BBB permeability could be studied in vitro using HBMEC or similar models. In addition, *Brucella* infection of microglia in vitro results in production of type I IFNs that activate STAT1 and induce nitric oxide release by microglia [25]. Nitric oxide then causes increased exposure of phosphatidylserine on cocultured neurons that in turn results in microglial phagocytosis of otherwise healthy neurons [26]. While type I IFN-dependent nitric oxide can damage neurons in vitro, we previously found IFN-γ–dependent nitric oxide suppresses *Brucella*-induced articular inflammation in vivo [16]. Therefore, how nitric oxide affects neuronal loss and inflammation in the CNS should be a topic of future study.

Overall, our work highlights the complex role of T cells, IFN, and STAT signaling during brucellosis. While the role of type I IFN in the presence of type II IFN may be masked, type I IFN signaling through both STAT1 and STAT2 in IFN-γ–deficient mice plays a crucial protective role that could be considered when investigating therapeutic interventions in cases of brucellosis, particularly as human brucellosis patients are more likely to have genetic polymorphisms associated with reduced IFN-γ production [27].
